# Microvascular Decompression Versus Stereotactic Radiosurgery for Trigeminal Neuralgia: A Decision Analysis

**DOI:** 10.7759/cureus.1000

**Published:** 2017-01-26

**Authors:** Ian Berger, Nikhil Nayak, James Schuster, John Lee, Sherman Stein, Neil R Malhotra

**Affiliations:** 1 School of Medicine, University of Pennsylvania School of Medicine, Philadelphia, PA; 2 Neurological Surgery, University of Pennsylvania School of Medicine, Philadelphia, PA

**Keywords:** trigeminal neuralgia, microvascular decompression, radiosurgery, gamma knife, cyberknife, decision analysis

## Abstract

Introduction: Both microvascular decompression (MVD) and stereotactic radiosurgery (SRS) have been demonstrated to be effective in treating medically refractory trigeminal neuralgia. However, there is controversy over which one offers more durable pain relief and the patient selection for each treatment. We used a decision analysis model to calculate the health-related quality of life (QOL) for each treatment.

Methods: We searched PubMed and the Cochrane Database of Systematic Reviews for relevant articles on MVD or SRS for trigeminal neuralgia published between 2000 and 2015. Using data from these studies, we modeled pain relief and complication outcomes and assigned QOL values. A sensitivity analysis using a Monte Carlo simulation determined which procedure led to the greatest QOL.

Results: MVD produced a significantly higher QOL than SRS at a seven-year follow-up. Additionally, MVD patients had a significantly higher rate of complete pain relief and a significantly lower rate of complications and recurrence.

Conclusions: With a decision-analytic model, we calculated that MVD provides more favorable outcomes than SRS for the treatment of trigeminal neuralgia.

## Introduction

Trigeminal neuralgia (TN) is a debilitating condition characterized by unilateral stabbing facial pain along the divisions of cranial nerve V. The lifetime prevalence of TN is estimated to be 0.7 per 1,000 people and usually presents between the fourth and seventh decades [1]. First line management involves medical therapy, although 10-25% of patients fail to respond to standard medication regimens and some patients may become resistant to medical treatment over time [2].  For patients with medically refractory TN, there are a number of proven invasive treatments. Approximately 8,000 patients undergo surgical treatment for TN in the U.S. annually with a total societal cost of over $100 million [3].  MVD is the most common surgical procedure performed for TN in the U.S.; however, the use of Gamma Knife (Elekta AB, Stockholm, Sweden) surgery has been increasing worldwide [4]. Unfortunately, many patients that undergo surgery experience a recurrence of symptoms after treatment, which further complicates the management of TN.

Microvascular decompression is a common nondestructive surgical approach used to treat medically refractory TN [5]. Approximately 80% of MVD patients experience immediate pain relief, with 75% and 64% maintaining relief after one year and 10 years, respectively [5]. However, age, degree of pain, medical comorbidies, previous procedures, or patient preferences may preclude the use of MVD [6]. Less invasive techniques such as SRS aim to lesion the nerve in order to achieve pain control. SRS avoids the operative risk of MVD and patients are usually discharged the same day. However, the pain recurrence rate is greater than 20% at five years, and the results appear less durable than MVD [7]. Other modalities of radiosurgery, such as linear accelerator-based radiosurgery, have been shown to be effective and safe in observational studies, but limited prospective data exists [8].

There is limited data to support treatment selection algorithms in choosing MVD or SRS in cases of clinical equipoise, where either procedure is a viable option [9]. Both surgeon and patient preferences preclude adequately powered trials. Only a few direct comparison studies between MVD and SRS exist in the literature, and none contain a random assignment of patients [7, 10-11]. In many studies, cohorts are operated on by a single surgeon, who likely has significant experience in that surgery. Complication rates from MVD are lower when the procedure is performed at a high-volume hospital or by a high-volume surgeon [12]. Additionally, there is much heterogeneity in reporting outcomes in TN [13]. Differences in pain scales and follow-up time hinder the direct comparison between studies and often leave the results open to interpretation. Commonly used outcomes such as pain-free and recurrence rates are influenced by complications. There are very few studies that report health-related quality of life (HRQoL) utility scores. If complication rates vary between MVD and SRS, then HRQoL should also differ between the procedures and may not be embodied by conventional metrics.

Here we present a decision analysis model for the treatment of trigeminal neuralgia comparing MVD and SRS using postoperative outcomes and complication data from the published literature. Our study aims to provide evidence for the selection of treatment, which will maximize quality of life for patients suffering from this disease.

## Materials and methods

### Model

**Figure 1:**
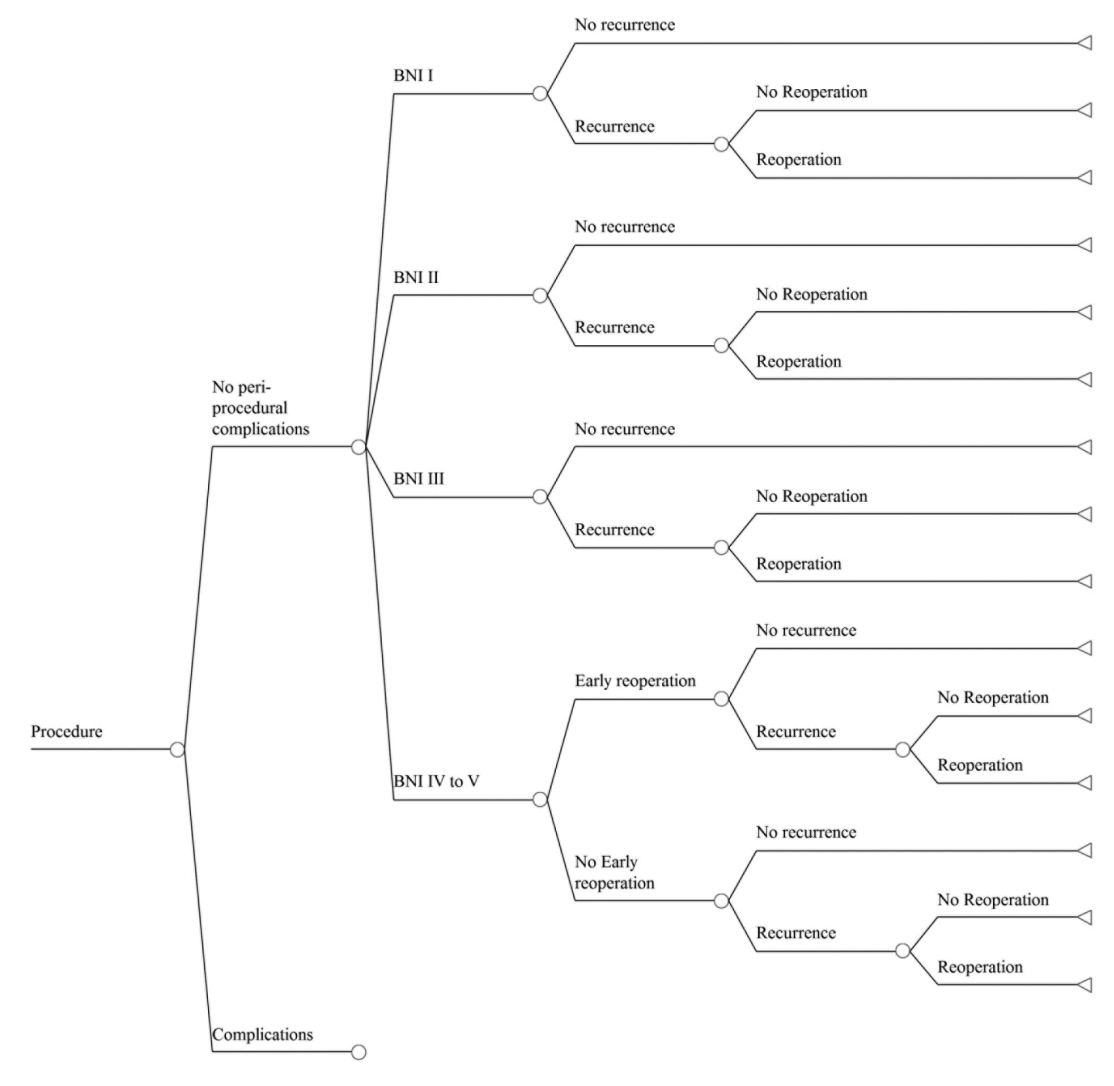
Outcomes of the Surgical Treatment of Trigeminal Neuralgia Decision tree outlining the possible pathways encountered by a patient undergoing either SRS or MVD for trigeminal neuralgia. Note that because of the very large number of branches contained in the entire tree, we only display outcomes of a single procedure, free of complications. The tree shown is repeated for cases in which complications occur, and this larger tree is repeated for the other procedure. The aggregate tree is then populated with the probability of each branch and the effect on quality of life for each outcome.

We constructed a decision-analytic model of outcomes following treatment for trigeminal neuralgia and related facial pain syndromes, using either MVD or SRS. The base case is a patient with uncontrolled pain despite maximal medical therapy. Reports in which atypical facial pain constituted a majority of the cases were omitted. The possible pathways and outcomes after treatment are shown in Figure 1. Either procedure can be associated with post-procedure complications or be complication free. We have modeled four possible pain outcomes, based on the Barrow Neurological Institute (BNI) Pain Intensity Score (Table 1). Once treatment occurs, the pain may recur (at a BNI level of III to V), and repeat treatment may be required.

**Table 1:** Barrow Neurological Institute Pain Intensity Score Patient reported pain intensity score for TN developed by the BNI [14].

Score	
I	No trigeminal pain, no medication
II	Occasional pain, not requiring medication
III	Some pain, adequately controlled with medication
IV	Some pain, not adequately controlled with medication
V	Severe pain/no pain relief

The model enters the probability of each possible outcome illustrated in Figure 1, as well as the effect of each outcome on HRQoL, as measured by utility [15]. A utility score is a parametric measure of a patient’s preference for a given health outcome in the face of uncertainty. A patient carries the utility associated with her/his BNI score, requirement or not of taking medications, and any complications associated with a procedure. The expected utility of each branch is found by multiplying the branch’s probability by the utility of the final outcome. Rollback analysis, the process that is used to calculate the expected utility of each branch and to add the resulting products for a given treatment strategy, produces the expected utility of that strategy. The expected utilities of two or more strategies thus represent their relative impacts on a patient’s QOL and are directly comparable.

Complications occurring in fewer than one percent of cases were omitted unless they had great impact on QOL (e.g. stroke and death). Transient complications, which have negligible effect on outcome, particularly after lengthy follow-up, are also not included. The model assumes that all cases in which pain recurs, does so after 2.5 years and has BNI scores of IV-V after pain returns. Patients who chose to re-treat their pain were limited to two additional procedures. Total quality-adjusted life years (QALYs) for the follow-up period were calculated by adding the QALYs of each year. For example, seven years in perfect health would equal seven QALYs.

### Literature search

We searched PubMed and the Cochrane Database of Sytematic Reviews for all publications between January 2000 and June 2015 for relevant articles. We performed multiples searches with the main subject headings “facial neuralgia” or “facial pain," plus either a single subheading or title term. The subheadings used were "treatment,” "surgery,” or “radiotherapy,” and the the title terms used were “microvascular,” “radiosurgery,” ”radiosurgical," “gamma,” “Linac,” “Cyberknife,” “Accuray," or “X knife.” A similar search was performed to determine the QOL corresponding to each outcome in the model. We limited our review to English language publications containing at least 10 treated cases.

### Data management

We abstracted estimates of complications, outcomes by BNI scores, utilities, recurrence, and reoperation rates from our search. Since most case series included patients with and without previous treatment, we included only publications in which fewer than 35% of cases had previous SRS or MVD. Not every article reported data for every category. Articles missing data for a category were excluded from the analysis of that category. Data was pooled meta-analytically [16] to calculate the probability of each outcome; the reported point estimates of data represent inverse variance-weighted means, which were tested to exclude heterogeneity [17]. The types and frequencies of complications are different after SRS and MVD. Since this is also true for several other parameters, we calculated their expected utilities using sub-trees. We examined outcomes at one- and seven-year follow-ups. Although some MVD studies follow patients for more than ten years, no SRS publication averages much longer than seven years. Recurrence rates, which vary with duration of follow-up, were determined by meta-regression for each follow-up time studied [18]. Meta-analysis and meta-regression employ random effects models. To factor in the effect of follow-up time, we reported outcomes at various follow-up durations as QALYs, a measure that combines both aspects [19]. Perfect health (QOL = 1) times one year of life equals one QALY; likewise, QOL of 0.25 times four years of life equals one QALY.

### Analysis

We used data obtained by literature review and pooled as above to populate our decision tree. Our primary analysis compared the two treatments with respect to expected QALYs after primary treatment. We performed sensitivity analysis using a two-dimensional Monte Carlo simulation (expected QALYs for 100 simulated trials, each made up of 100 microsimulations) [20]. We employed beta distributions for all probabilities and utilities. We calculated pooled demographic data, such as mean ages, gender distribution, and previous procedures, for the two treatment groups.

Because the events after MVD and SRS occur at different times after treatment, the model assumes that the post-treatment BNI state occurs immediately after MVD but, on average, at one month after SRS. Re-treatment for therapeutic failures occurs at one and three months after primary treatment for MVD and SRS, respectively. The wait is to allow for possible delayed effects of the initial therapy. Similarly, treatment complications are assumed to occur immediately after MVD and an average time of six months after SRS. Keeping with standard decision-analytic practice, QOL associated with multiple outcomes is calculated by multiplying the utilities of each [21]. For example, in a subject with BNI III and surgical complications after MVD, the expected utility would equal the utility of MVD complications times the utility of BNI III times the utility of requiring medication. Random effects meta-regression models were used to investigate recurrence rates and relationships between treatment type, demographic variables (age, gender, length of follow-up, and previous pain procedures), and outcome. Since it was impractical to run the decision-analytic model separately for each case series and calculate QALYs associated with each, we elected to use the percentage of cases with post-treatment BNI scores of I as our primary measure of successful outcome. We also looked at rates of failure (BNI score = IV-V), complications, and recurrence.

Comparisons of expected utilities at each time point used t-tests, and probabilities of < 0.05 were considered significant. Meta-analytic pooling, meta-regressions, and statistical analyses were done with inverse-variance-weighted random effects models using Stata 12 (StataCorp LP, College Station, TX). Analyses of the model, including Monte Carlo simulation, employed TreeAge Pro 2012 (TreeAge Software, Inc., Williamstown, MA).

## Results

### Literature search

**Figure 2:**
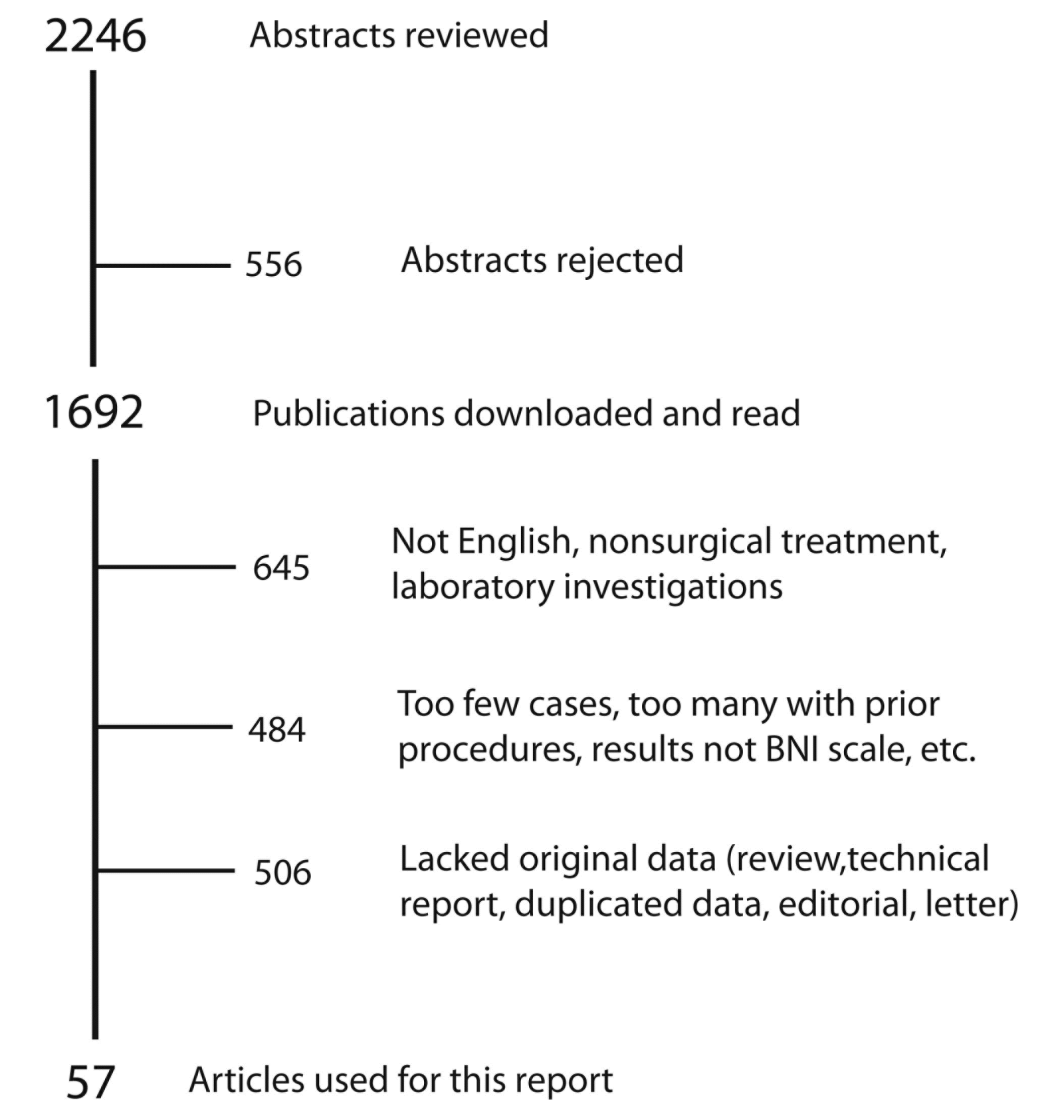
Articles Reviewed Summary of abstracts and articles reviewed for this report. Reasons for the rejection of case series are shown.

The search yielded 2,246 publications. After rejecting unsuitable abstracts and excluding publications without useful data, there were 57 publications including 8,484 cases remaining, which form the basis for our analysis. Search results are shown in Figure 2. The characteristics of these studies are presented in Table 2.

**Table 2:** Publications Used in This Analysis Publications used in the analysis arranged by publication date.

First Author	Type of Procedure	Number of Cases	Study Type
Brisman [22]	SRS	49	Observational
Nicol [23]	SRS	42	Observational
Chang [24]	SRS	15	Observational
Shaya [25]	SRS	40	Observational
Lim [26]	SRS	41	Observational
Tawk [27]	SRS	38	Observational
Pollock [28]	Both	83	Prospective Cohort study
Massager [29]	SRS	109	Observational
Fountas [30]	SRS	77	Observational
Balamucki [31]	SRS	172	Observational
Fountas [32]	SRS	57	Observational
Longhi [33]	SRS	92	Observational
Brisman [34]	SRS	61	Observational
Lorenzoni [35]	SRS	89	Observational
Matsuda [36]	SRS	100	Observational
Huang [37]	SRS	89	Observational
Linskey [11]	Both	70	Prospective Cohort study
Oh [38]	Both	45	Retrospective comparison
Dhople [39]	SRS	112	Observational
Knafo [40]	SRS	67	Observational
Han [41]	SRS	51	Observational
Fariselli [42]	SRS	33	Observational
Adler [43]	SRS	46	Observational
Park [44]	SRS	39	Observational
Chen [45]	SRS	40	Observational
Sheehan [46]	SRS	63	Observational
Hayashi [47]	SRS	130	Observational
Park [48]	SRS	17	Observational
Park [49]	SRS	62	Observational
Tuleasca [50]	SRS	497	Observational
Fraioli [51]	SRS	23	Observational
Attia [52]	SRS	19	Observational
Lee [53]	SRS	91	Observational
Flickinger [54]	SRS	174	Observational
Lazzara [55]	SRS	16	Observational
Young [56]	SRS	250	Observational
Lee [57]	SRS	21	Observational
Lucas [58]	SRS	446	Observational
Song [59]	SRS	20	Observational
Xu [60]	SRS	99	Observational
Tyler-Kabara [61]	MVD	1739	Observational
Ashkan [62]	MVD	80	Observational
Olson [63]	MVD	156	Observational
Zakrzewska [64]	MVD	220	Observational
Aryan [65]	MVD	19	Observational
Sindou [6]	MVD	362	Observational
Laghmari [66]	MVD	51	Observational
Tarricone [67]	MVD	20	Observational
Bond [68]	MVD	119	Observational
Oesman [69]	MVD	120	Observational
Chakravarthi [70]	MVD	40	Observational
Chen [71]	MVD	67	Observational
Zhong [72]	MVD	1274	Observational
Sandel [73]	MVD	243	Observational
Reddy [74]	MVD	60	Observational
Shibahashi [75]	MVD	65	Observational
Sarnvivad [76]	MVD	98	Observational

### Patient characteristics

Table 3 demonstrates the demographic differences between the two treatments. Compared to MVD, patients undergoing SRS are significantly more likely to be older. Follow-up in MVD patients is significantly longer. The trend of more SRS patients having undergone a previous facial pain procedure approaches significance, and sex distribution is the same in the two groups.

**Table 3:** Patient Characteristics

Characteristic	SRS			MVD			Difference (p-value)
	N	Mean	SD	N	Mean	SD	
% Female	3144	60.9	8.0	1699	62.7	8.0	0.462
Mean Age	1944	67.6	4.5	3697	57.5	3.3	<0.001
Mean Follow-up (mos)	3477	31.7	19.2	3697	43.2	27.6	0.065
% With Previous Procedures	3013	21.1	10.4	3387	14.9	10.8	0.058

### Comparative effectiveness

Probabilities and utilities of possible outcomes for the tree (Figure 1) are summarized in Table 4 and Table 5, respectively. Cure of pain (BNI score of I) is significantly more likely after MVD; poor pain control, complications, and recurrences are significantly lower after MVD than SRS. The sorts of complications encountered after either SRS or MVD are shown in Table 6, along with the utility of each. When complications occur, their impact on QOL does not depend on surgical approach (p = 0.850).

**Table 4:** Probabilities of Outcomes After Treatment

Outcome		SRS		MVD		Difference (p-value)
		Mean	SD	Mean	SD	
BNI score (%)						
	I	38.8	17.5	52.8	21.3	0.01
	II	20.9	10.8	26.2	24.5	0.256
	III	25.5	12.7	13.3	5.1	<0.001
	IV-V	14.8	6.4	7.7	0.67	<0.001
Complications (%)		19.3	0.7	17.6	0.5	<0.001
7-year recurrence rate (%)		22.6	13.4	15.9	10.5	0.013
Recurrences re-treated (%)		45.5	35.5	8.0	6.9	0.005

**Table 5:** Utilities of Outcomes After Treatment of Trigeminal Neuralgia by MVD or SRS N/A = not appropriate

Description	Utility	SD	Ref
BNI I	1.0	N/A	Hunink 2001 [21]
BNI II	0.871	0.213	Perez et al. 2009 [77]
BNI III	0.739	0.221	Perez et al. 2009 [77], Spatz et al. 2007 [78]
BNI IV-V	0.399	0.189	Perez et al. 2009 [77]
Complications after MVD	0.958	0.013	Calculated from analysis (Table 7)
Complications after SRS	0.962	0.085	Calculated from analysis (Table 8)
Needing to take medication	0.881	0.003	Calculated from analysis (Table 9)
Re-treatment by MVD	0.915*(utility MVD)	N/A	Lega et al. 2010 [79]
Re-treatment by SRS	0.915*(utility SRS)	N/A	Lega et al. 2010 [79]

**Table 6:** Effect of Periprocedural Complications on Utility CSF = cerebrospinal fluid; NR = not reported

Name	Mean Utility	SD	Ref
No complications	1.0	0	Hunink 2001 [21]
CSF leak	0.985	NR	Estimate
Facial palsy	0.983	0.018	Whitmore et al. 2011: Vestibular schwannoma [80]
Corneal keratitis	0.963	NR	van de Graaf et al. 2010 [81]
Facial numbness, paresthesia,​ or dysesthesia	0.960	0.014	Whitmore et al. 2011: Vestibular schwannoma [80]
Meningitis	0.930	0.030	Whitmore et al. 2011: Vestibular schwannoma [80]
Unilateral deafness	0.929	0.110	Whitmore et al. 2011: Vestibular schwannoma [80]
Acute subdural hematoma	0.868	.0170	Lega et al. 2010: Chronic SDH [79]
Diplopia	0.762	0.104	Hatt et al. 2010 [82]
Stroke	0.5	NR	Samsa et al. 1999 [83]
Perioperative death	0	0	Hunink 2001 [21]

**Figure 3:**
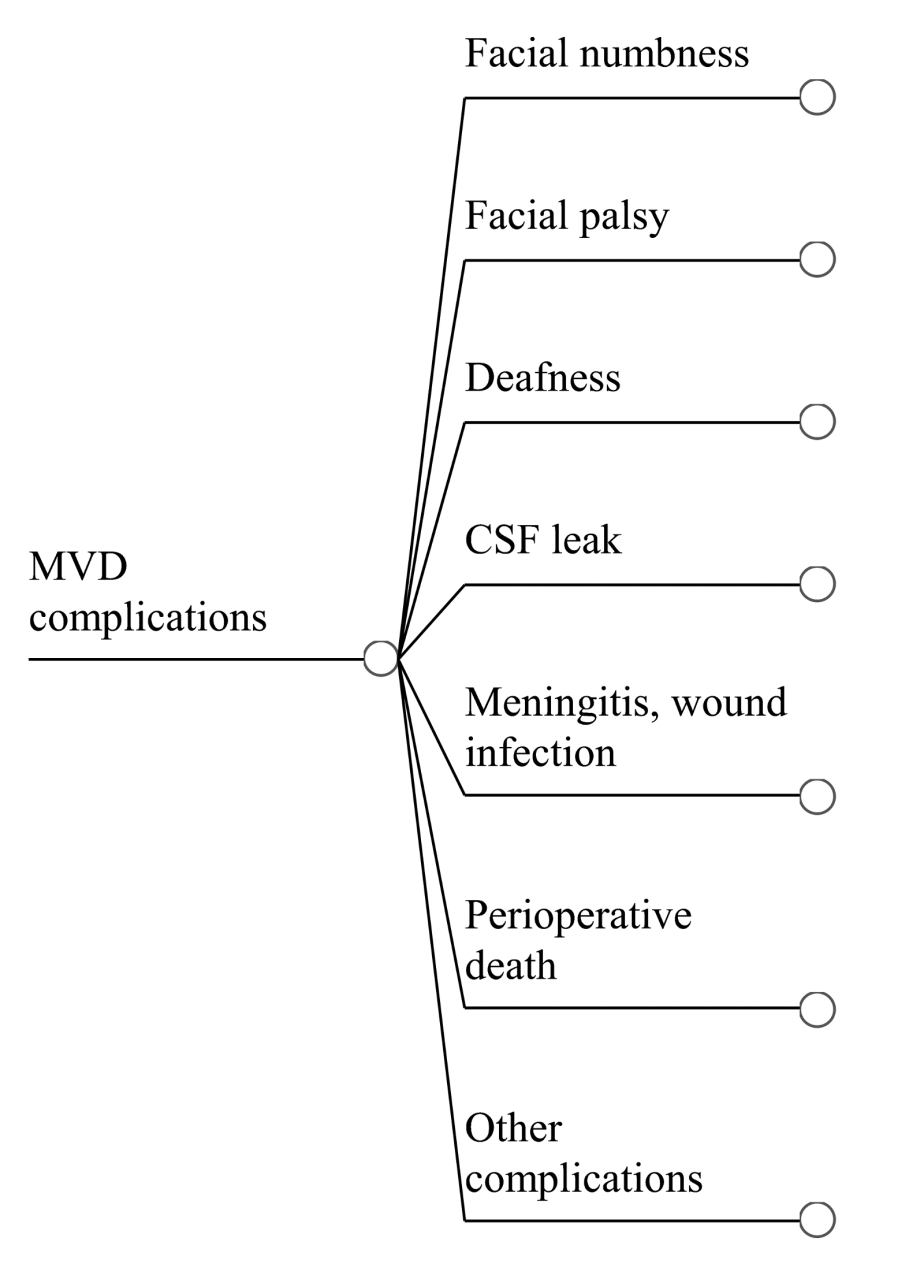
Complications of MVD Treatment Sub-tree outlining complications reported after MVD for trigeminal neuralgia.

Table 7 shows complication rates following MVD. Individual procedure-related complications are assumed to add. The total perioperative complication rate is 13.3%. With the decision tree shown in Figure 3, the expected utility of patients with complications after MVD is 0.958 ± 0.013.

**Table 7:** Incidence of Complications After MVD for Trigeminal Neuralgia CSF = cerebrospinal fluid

Complication	Mean	SD
Facial numbness	0.044	0.020
Facial palsy	0.011	0.017
Perioperative death	0.002	0.001
Deafness	0.018	0.030
CSF leak	0.013	0.021
Meningitis, wound infection	0.012	0.016
Other complications	0.033	0.005

**Figure 4:**
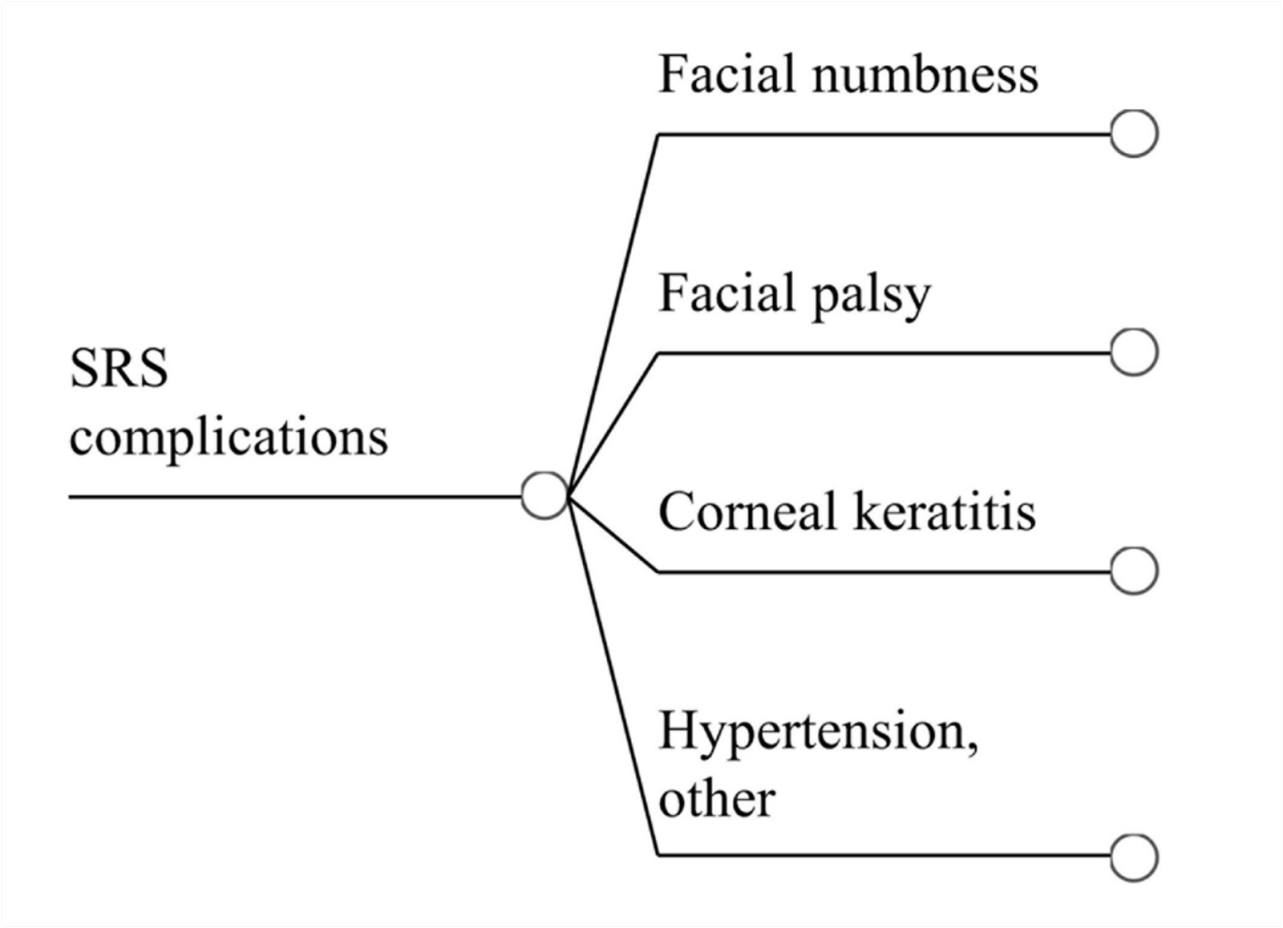
Complications of SRS Treatment Sub-tree outlining complications reported after SRS for trigeminal neuralgia. Populating the tree with the probability and utility associated with each complication permits calculation of expected utility in patients who suffer complications after SRS.

Table 8 summarizes the complication rates for patients undergoing SRS for TN. The sole exception to the addition of individual complications is keratitis, in which patients are assumed to also have trigeminal and facial nerve deficits. The total procedure-related complication rate is 19.3%. Figure 4 is a decision tree to calculate expected utility for patients encountering complications after SRS, which was found to be 0.962 ± 0.085.

**Table 8:** Incidence of Complications After SRS for Trigeminal Neuralgia

Complication	Mean	SD
Facial numbness	0.141	0.053
Facial palsy	0.017	0.013
Corneal keratitis	0.015	0.009
Hypertension, other complications	0.020	0.019

**Figure 5:**
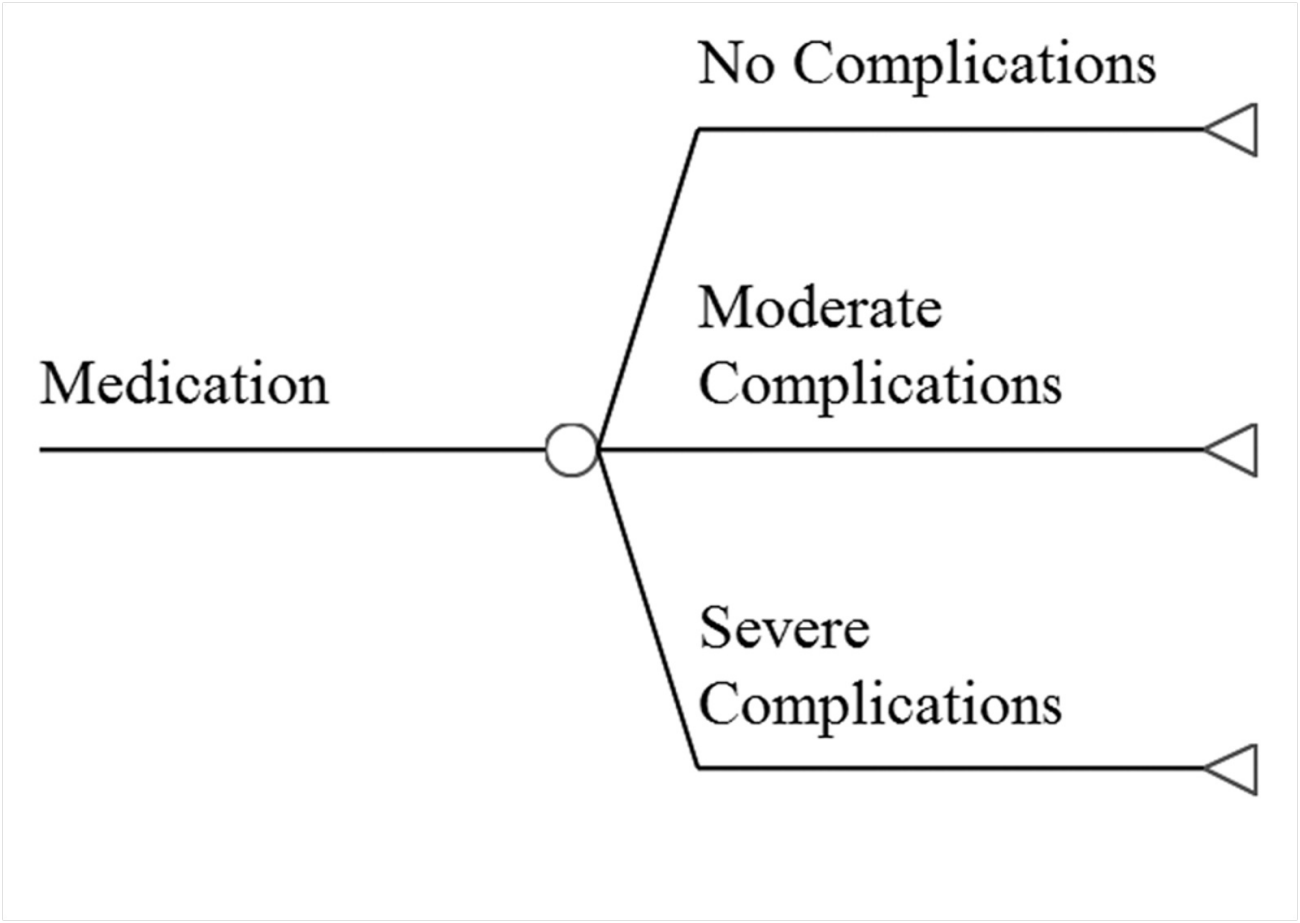
Outcomes of Medical Treatment Sub-tree for calculating the expected utility of taking maintenance medication.

Taking medicine several times daily has a modest impact on QOL. However, the presence of complications associated with its use can have severe repercussions. Table 9 reports probabilities and associated utilities of different severities of medication complications. Analysis of the decision tree (Figure 5) employing these values yielded an expected utility of 0.881 ± 0.003.

**Table 9:** Probability and Utility of Trigeminal Neuralgia Medication Side Effects Values based on a decision analysis by Spatz et al. examining which treatments for TN offer the best patient quality of life [78].

Description		Value	SD
Probability			
	no medication complications	0.176	0.092
	mild–moderate medication complications	0.514	0.144
	severe medication complications	0.311	0.126
Utility			
	no medication complications	0.940	0.010
	mild–moderate medication complications	0.912	0.037
	severe medication complications	0.774	0.148

Meta-regressions of recurrence rates after SRS and MVD failed to suggest an increasing recurrence rate with the passage of time (p = 0.597 and 0.492, respectively). Meta-analysis was therefore used to calculate seven-year recurrence rates for SRS and MVD. The results are reported in Table 4. The model assumes that all recurrences occur midway in the follow-up period, and pain recurrences not requiring surgical (radiosurgical) procedures are all BNI level III. Treatment modality for failure of primary treatment or recurrence is based on the proportions of procedure types reported in the literature. SRS cases with recurrence tended to involve MVD for recurrences; failed MVD cases were followed by a second MVD in 80% of cases. The small number of percutaneous reoperations were not included in the analysis, as they had minimal impact on outcome.

We calculated expected QOL at seven years after the pain procedure. MVD outcomes (6.009 ± 0.173 QALYs) are significantly higher (p < 0.001) than those with SRS (5.411 ± 0.132 QALYs). The higher utility of MVD is attributable in part to its higher initial success rate and lower pain recurrence rate compared to SRS.

Meta-regression of outcomes against demographic factors revealed only a few near-significant correlations shown in Table 10. For MVD patients, success (post-procedure BNI I) was inversely related to age. There was a near-significant inverse trend in the SRS group between prior procedures and success, and a trend in MVD patients between prior procedures and complication rate. Recurrence rate did not increase significantly over time after either procedure.

**Table 10:** Associations Among Predictor Variables and Outcomes Significant associations shown in bold. ^1^Success = BNI score I ^2^Failure = BNI score IV-V R^2^ = coefficient of determination

Predictor variable		Outcome variable							
		success rate^1^		failure rate^2^		complication rate		recurrence rate	
		R^2^	p-value	R^2^	p-value	R^2^	p-value	R^2^	p-value
SRS									
	Age	< 0.01	0.599	< 0.01	0.906	< 0.01	0.820	< 0.01	0.768
	Sex	0.012	0.248	0.023	0.584	0.024	0.210	0.029	0.585
	Follow-up (mos)	not measured							
	Prior Procedure	0.074	0.051	0.001	0.353	0.106	0.071	0.075	0.108
MVD									
	Age	0.99	0.023	0.485	0.410	< 0.01	0.908	< 0.01	0.884
	Sex	0.034	0.649	0.059	0.901	0.099	0.901	< 0.01	0.524
	Follow-up (mos)	not measured						0.022	0.492
	Prior Procedure	0.070	0.741	0.027	0.500	0.206	0.056	0.126	0.267

## Discussion

TN commonly becomes refractory to medication and may require surgical intervention. There are many studies reporting successful outcomes of various procedures for TN; however, the subjective nature of pain and lack of HRQoL data make comparisons difficult. The results of this study suggest MVD is significantly more likely to produce a BNI I outcome and result in greater QOL than SRS at a seven-year follow-up. MVD had significantly less complications and a lower recurrence rate at seven years. MVD success rates were inversely correlated with age.

### Microvascular decompression surgery

Microvascular decompression is currently the most common surgical treatment for TN [84]. MVD procedures in the U.S. increased 194% between the years of 1988 to 2008 [85]. While considered safe, MVD is a major neurosurgical procedure that carries some risk.

Many observational studies have found MVD to be an effective treatment for TN. Tatli et al. [86] conducted a review of all surgical treatments for TN, including 16 studies for MVD in which the average follow up was 6.7 years. Pain-free rates at last follow-up were 76.6%, and the average recurrence rate was 18.4% at last follow-up. They also found the rates of facial numbness at one to two percent. Our analysis found that MVD resulted in a lower rate of BNI I outcomes (52.8% at seven years). However, Tatli et al. noted that the results of many of the observational studies were up to interpretation. Our rate of BNI II was very high (26.2%), and it is possible that this group of patients was included in the “treatment success” group in Tatli’s review. We found a recurrence rate of 15.9%, which is similar to the 18.4% reported by Tatli [86]. Recurrence can occur because of compression of the nerve from the interposing material placed during surgery or recontact of a vessel with the nerve. A “sling” technique may be used to hold the vessel off the nerve, and it demonstrates promise at lowering recurrence rates despite its technical difficulty [87]. The most common reported complication of MVD in our study was facial numbness, affecting 4.4% of patients. This rate is higher than that reported by Tatli [86]. Some of our included studies did not distinguish between permanent numbness and transient or non-bothersome numbness, which may explain the higher rate. Our model found that complications from MVD were significantly higher if the patient had undergone a previous procedure, which is consistent with previous reports in the literature [88-89]. In a study of 305 hospitals from 1996 to 2000, it was reported that the mortality rate for MVD was 0.3%, similar to 0.2% found in our study [12]. Mortality was associated with surgeons who performed relatively few MVDs.

MVD is an open surgical procedure that is associated with complications thought to be greater in elderly populations [90]. However, recent studies have shown that MVD can be just as effective and safe for elderly patients [62]. We found that age was inversely related to a BNI I after MVD. Thus, while age cannot be ruled out as an independent predictor of outcome for MVD, SRS may be the more effective treatment in the elderly.

Compared to SRS, MVD was shown to have better outcomes with regard to complete pain relief, lower complication rates, and recurrence rates. Additionally, the mean follow-up time for MVD studies, while not statistically significant, was longer, providing some evidence for the durability of the results. New techniques such as endoscopic MVD are reaching clinical practice and may further improve the safety of MVD for TN, although further outcomes research with endoscopic techniques are still needed [91].

### Stereotactic radiosurgery

Radiosurgery has evolved dramatically with advancements in imaging allowing more precise targeting, devices allowing better delivery that spares the brainstem, and better clinical data to plan the most effective dose. There is a wide range (22-83%) in the rates of pain-free outcomes from SRS reported in the literature [92-93]. This variability may have to do with the different follow-up periods of these studies, as outcomes from SRS have been shown to be much less durable than MVD [27]. According to a study by Pollock et al. [94], the risk of trigeminal nerve dysfunction is related to the dose of radiosurgery, and the author found that 50% of patients receiving a dose of 90 Gy experienced new dysfunction compared to 15% of those receiving 70 Gy. In a second study, he associated new dysfunction to decreasing treatment success rates [95]. Massager et al. noted that patients receiving higher doses of radiation to the brainstem experienced increased rates of facial numbness, although this was not significant in their study [96]. However, increased radiation doses delivered to the brainstem significantly predicted pain control.

Several studies have investigated the role of anterior or posterior targeting of the trigeminal nerve during SRS treatments. Matsuda et al. [36] found that targeting the dorsal root entry zone (DREZ) compared to the nerve proximal to the gasserian ganglion produced higher initial rates of pain relief and lower complication rates. However, Park et al. [44] found a decrease in time to response when targeting the retrogasserian zone compared to the DREZ. No differences in complication rates were found in this study. Within the DREZ, proximal targeting of the nerve may lead to decreased rates of recurrence, but an increased rate of new facial numbness [60]. While the use of one versus two isocenters does not seem to have an effect on pain relief, higher, but not statistically significant, rates of sensory loss were found in patients treated with two isocenters in a prospective, blinded trial performed by Flickinger et al. [97]. However, a separate study demonstrated increases in pain relief using two isocenters without increases in complication rate [98].

Parmar et al. noted in their review that, although SRS complication rates are significant, they have been improving over time, perhaps related to advances in imaging and dose delivery [99]. MRI images that use the thinnest slices and highest resolution lead to the most accurate surgeries with Gamma Knife [100]. Recurrence rates in the literature are between six and 41% [101]. Patients that experience a recurrence in pain may undergo a second round of radiosurgery but at an increased risk to developing facial numbness [102].

The results of our study are consistent with the literature on outcomes for SRS. Our mean complete pain relief rate was 38.8%, while we calculated a 22.6% recurrence rate at seven years. The largest morbidity for SRS in our study was facial numbness at 14.1%. Cruccu et al. found, in a review for the American Academy of Neurology and European Federation of Neurological Societies (AAN-EFNS) guidelines, that facial numbness affects nine to 37% of patients in large studies [103], which was consistent with our findings. The higher rates of morbidity and recurrence limit the utility of SRS in treating TN. In our study, we found that SRS patients were older, and there was a trend toward significance of having had prior procedures. This finding is consistent with the often held view that SRS is reserved for older patients with recurrent pain. Due to its minimally invasiveness, it may be used to treat TN refractory to MVD [104]. However, the Maesawa et al. study noted that prior procedures were associated with a lower rate of complete pain relief [105].

The timing of re-treatment of TN with SRS after initial treatment failure is a topic of discussion. Initial pain-free rates increase dramatically within the first three months after treatment before plateauing at six months [106]. Thus, the decision to re-treat should be done at least six months, and often one year, after initial therapy. Our model cannot capture the variability that occurs in clinical practice and the uncertainty of treatment success that exists between therapy and the six-month decision to treat. Patients who are intially pain free may experience a brief relapse of pain that eventually dissipates. Therefore, in our model, patient response to therapy was determined at one month after treatment, and the decision to re-treat was done at three months. Furthermore, there is a subset of patients who are initially pain free that may develop a recurrence of pain, which then gradually subsides. This pathway was not included in our analysis, given the complexities of incorporating these patients into the model and the lack of literature on these patients.

### Comparative studies

Several studies have compared the cost effectiveness of the surgical treatments for TN. Both Holland et al. [107] and Pollock et al. [3] conducted small studies in which they found that MVD produced a higher number of quality-adjusted pain-free years and was more cost effective than SRS. Sivakanthan et al. found that MVD produced a QALY gain of 8.2 at a five-year follow-up, while SRS was 4.9 QALYs gained [84]. With MVD at 6.0 and SRS at 5.4 QALYS, our study demonstrated a smaller discrepancy in QALYs gained than Sivakanthan. However, Sivakanthan’s study only used one article each to calculate the QALYs for the treatments. Spatz et al. performed a decision analysis in 2007 comparing medical therapy, MVD, percutaneous glycerol rhizolysis, radiofrequency thermocoagulation, and balloon compression, using a cohort of 156 patients [78]. They concluded that MVD was the procedure most likely to maximize QOL; however, SRS was not included as a treatment modality.

Two studies have looked at the impact of MVD and SRS on patient satisfaction and found that the durability of pain relief with MVD greatly influences patient satisfaction with the procedure compared to SRS [7, 11]. However, they used a small number of patients, and adequate literature on patient satisfaction is lacking.

### Quality of studies and limitations

The overall quality of the studies used in the decision analysis was generally poor when assessed individually. This had previously been noted in 2003 by Zakrewska et al. [108] and continues to hold true. Many articles used their own scales for treatment success and pain relief, leaving the results open to interpretation and hindering comparison. The overwhelming majority of the articles used in this analysis were observational, demonstrating the lack of randomized controlled trials in the literature for surgical treatments of trigeminal neuralgia. Most of the studies provided a description of the patient characteristics; however, preoperative pain, sensory loss, and medication dosages were frequently not included. Both outcome measures and complication rates were heterogeneous and in some instances, only vaguely identified. However, most studies reported recurrence rates within their follow-up window.

If each publication reported utility scores for patients before and after treatment, a direct comparison of utility change after SRS and MVD would be possible. Unfortunately, these scores are rarely obtained. Instead, our model assumes that a complication-free procedure, resulting in a BNI score of I, has a utility of one. Although the resulting utilities are not equivalent to scores reported in actual TN patients [77, 109], the relative utilities of the treatment groups remain the same. Thus, the calculated utilities accurately reflect the relative outcomes and permit valid statistical comparisons.

Our results must be interpreted with caution. The model makes several simplifying assumptions, any of which can impact our results. Our data arise from a series of indirect, nonrandomized comparisons, within which there is clinical heterogeneity. The studies analyzed varied with respect to patient selection, baseline severity of pain, treatment assignment, outcome parameters measured, and time of follow-up. The use of a random effects model partially compensates for heterogeneity but cannot completely correct for it. For example, the majority of TN studies fail to record the preoperative level of pain, preventing a comparison between pre- and post-treatment [108]. While there are clinical pain scales to correct this problem, they were not used in our analysis [110]. Our reliance on trial-level data, rather than individual patient information, may also introduce aggregation bias.

In addition to Monte Carlo simulations, we ran sensitivity analyses in which we systematically varied values of each major variable. None had a significant impact on our results except when varied to extreme values. We cannot rule out age as an independent predictor of outcome in MVD, suggesting SRS may be the more effective treatment in the elderly. There are near-significant trends linking prior procedures with lower success rates with SRS and with higher complication rates after MVD.

There are other limitations to our study. The HRQoL data we used is taken from the literature as a proxy to create a TN disease state rather than from individual patients. The values for perioperative complications are from different sources and are not specific to TN. Even so, our model assumes that the relative values can be compared between MVD and SRS. Additionally, complications in the literature are presented dichotomously and don’t represent the duration or severity that could impact the measurement on QOL. For example, facial numbness was tracked in most of the studies, but the degree and distribution of numbness was not recorded and could have a variable impact on the treatment utility. Finally, surgery for medically refractory TN cannot be determined by a standard algorithm. Heterogeneity among patients precludes true clinical equipoise. Many of the included studies had cohorts that were operated on by a single surgeon. The effect of surgeon experience on outcome should not be underestimated in procedures such as MVD and cannot be accounted for by our model.

The results of this study aim to direct surgical decision-making for the treatment of medically refractory TN in cases where MVD and SRS are both suited. While surgeon and patient preference play a large role in decision-making, this study suggests that MVD provides a higher QOL than SRS, and that initial pain-free rates, recurrence rates, and complications play a large role in postoperative HRQoL. This study, and the observational studies it draws from, demonstrates the need for standardized, high-quality research to continue to explore the application of surgery to TN.

## Conclusions

MVD for trigeminal neuralgia is associated with a significantly higher quality of life than SRS. This difference is primarily attributable to MVD’s higher initial cure rate and lower recurrence rate. Although follow-up data for SRS does not permit comparison after seven years of follow-up, the trend toward rising recurrence rates following SRS suggests that the advantages of MVD are more durable.
